# Pre-hospital assessment of trauma associated severe hemorrhage (phTASH) – analysis of TraumaRegister DGU^®^ data from 2015-2021

**DOI:** 10.1186/s13049-025-01404-4

**Published:** 2025-07-01

**Authors:** Christoph Jänig, Willi Schmidbauer, Erwin Kollig, Tobias Gruebl, Rolf Lefering, Lisa Hackenberg, Daniel C. Schroeder, Dan Bieler, Committee on Emergency Medicine, Intensive Care, Trauma Management (Sektion NIS) of the German Trauma Society (DGU)

**Affiliations:** 1 Department for Anesthesiology and Critical Care Medicine, German Armed Forces Central Hospital Koblenz, Rübenacher Str. 170, 56072 Koblenz, Germany; 2 Department for Trauma Surgery and Orthopedics, Reconstructive Surgery, Hand Surgery, Burn Medicine, German Armed Forces Central Hospital Koblenz, Rübenacher Str. 170, 56072 Koblenz, Germany; 3 Institute for Research in Operative Medicine, Witten/ Herdecke University, Ostmerheimer Str. 200, Building 38, 51109 Cologne, Germany; 4 Department of Anaesthesiology and Critical Care Medicine, Faculty of Medicine and University Hospital of Cologne, University of Cologne, Kerpener Str. 62, 50937 Cologne, Germany

**Keywords:** Hemorrhage, Emergency medical services, Massive transfusion, Polytrauma

## Abstract

**Background:**

Prehospital transfusions are becoming increasingly popular in Europe. Blood products play an important role in the treatment of massive hemorrhage but may pose a potential risk to the patient when the indication is inconclusive such as in a prehospital setting. Simple scores are required in order to initiate targeted therapy and to use the valuable resource of blood comprehensibly in the prehospital setting. With the Assessment of Blood Consumption (ABC)-score and the reversed Shock Index GCS (rSIG)-score, two scores with a good predictive quality for the occurrence of massive transfusion have already been described. However, both scores were validated with in-hospital data that do not correspond to the characteristics of the European trauma population. Aim of this study is to validate both scores with data that represent the European trauma population and to discuss the usability to justify prehospital blood transfusion.

**Methods:**

Using data from the TraumaRegister DGU^®^ from the years 2015–2021, this study examines the proportion of patients who required a transfusion or massive transfusion after hospital admission. The results are used to calculate the area under the receiver operating characteristics curve (AUROC) to assess the predictive quality of the ABC score and the rSIG score for an European trauma population. A logistic regression analysis is used to identify the items of both scores with the highest impact on the predictive quality of each score. The items with the most influence were then combined to form the new prehospital Trauma Associated Severe Hemorrhage (phTASH) score.

**Results:**

We included 63,946 datasets. 8.5% of all patients received a transfusion and 1% a mass transfusion after hospital admission. The mean Injury Severity Score (ISS) for patients with massive transfusion was 39.9 vs. 16.8 without massive transfusion. The ABC score has an AUROC of 0.711 (0.702–0.719) for any transfusion (≥ 1 packed Red Blood Cells; pRBC) and of 0.806 (0.786–0.826) for massive transfusion (≥ 10 pRBC). The rSIG score has an AUROC of 0.737 (0.730–0.744) vs. 0.807 (0.790–0.824) respectively. The phTASH score is calculated with an AUROC of 0.747 (0.737–07.53) for the prediction of any transfusion and an AUROC of 0.834 (0.816–0.952) for the prediction of massive transfusion after severe trauma. The three items with the greatest influence on the score results are the positive Focused Assessment with Sonography in Trauma (FAST) examination (OR 5.28), systolic blood pressure < 90mmHg (OR 2.94) and a shock index > 1 (OR 2.49).

**Conclusions:**

The need for transfusion can easily be assessed even in the prehospital setting. The new developed phTASH score has a high predictive accuracy for transfusions after trauma. The identification of a positive FAST examination as the element with the greatest impact on predicting a patient’s need for transfusion emphasizes the importance of prehospital ultrasound.

## Background

Blood products are a valuable resource in the treatment of acute hemorrhage following trauma. Blood products must be used in a sensible and individually targeted manner to avoid unnecessary resource allocation and minimize the risk of unnecessary transfusion reactions.

The preclinical stockpiling and application of blood products is taking place on an increasingly large scale, even if the relevance to the outcome of patient care cannot be assessed reliably yet [1–3]. 

The early use of blood products has a positive influence on patient condition and survival in the early phase after a severe trauma, which justifies prehospital or at least early hospital transfusion in the trauma resuscitation phase [4, 5]. Furthermore, the time factor also appears to be decisive regarding the evidence [6–8]. 

There are multiple scores to predict the need for massive transfusion in trauma [9]. To identify patients with a potential need for early transfusion, we initially chose two different scores to identify items, that are easily available in the prehospital setting and documented within the datasets of the TraumaRegister DGU^®^ (TR-DGU) to conduct this study. The first score - that was also used by Frieler et al. to predict mortality after trauma [10]- is the “Reverse shock index multiplied by Glasgow Coma Scale score” (rSIG)-score, consisting of the systolic blood pressure divided through heart rate and multiplied with the **G**lasgow-Coma-Scale value [rSIG= (systolic blood pressure/heart rate)*Glasgow Coma Sscale] on scene taking hemodynamic impairment and resulting organ dysfunction into account. In comparison to other known prediction scores for massive transfusions, the rSIG score can already be reliably determined preclinically. With an area under the receiver operating characteristics curve (AUROC) of 0.84 (95% CI 0.72–0.81) it also has good predictive quality (sensitivity 0.79, specificity 0.77) at a Cut-off value of 9.51 [11]. 

As a second score we used the Assessment of Blood Consumption (ABC) score to estimate the need for early transfusion. The ABC score is calculated out of the following items: (1) systolic blood pressure < 90 mmHg, (2) heart rate > 120 bpm, (3) positive FAST exam and (4) penetrating injury to the thorax. With a described AUROC of 0.763 (95% CI 0.732–0.794; sensitivity 76.1%, specificity 70.3%) at a cut off value of 2 points, it also provides a reasonable predictive quality and is described as a score that is suitable for the prehospital setting [12]. 

Using data from the TraumaRegister DGU^®^ (TR-DGU) from 2015 to 2021, this study examines whether it is possible to make a sufficient preclinical assessment of transfusion requirements after trauma using a score that is easy to be applied.

In detail, a prehospital transfusion score could be used to initiate a prehospital transfusion. Further, the scoring could be forwarded to the receiving hospital that prepares and orders blood products respectively to be able to initiate transfusion immediately after admission [13]. 

The aim of this study is therefore to identify a score that is easy to collect preclinically with a good predictive quality to either justify an immediate transfusion or to be able to carry out a valid pre-registration. The develpoement of the new phTASH score resulted after an initial analysis of the two above mentiones scores and after identification of the most important items from both scores.

## Materials and methods

### TraumaRegister DGU^®^

The TraumaRegister DGU^®^ (TR-DGU) of the German Trauma Society (Deutsche Gesellschaft für Unfallchirurgie, DGU) was founded in 1993. The aim of this multi-center database is a pseudonymised and standardised documentation of severely injured patients.

Data are collected prospectively in four consecutive time phases from the site of the accident until discharge from hospital: (A) Pre-hospital phase, (B) Emergency room and initial surgery, (C) Intensive care unit and (D) Discharge. The documentation includes detailed information on demographics, injury patterns, comorbidities, pre- and in-hospital management, course on the intensive care unit (ICU), relevant laboratory findings including data on transfusion, and outcome of each individual. The inclusion criterion is admission to hospital via the emergency room (trauma team activation) with subsequent intensive or intermediate care. Patients who reached the hospital with vital signs but died before admission to ICU were included as well.

The infrastructure for documentation, data management, and data analysis is provided by AUC - Academy for Trauma Surgery (AUC - Akademie der Unfallchirurgie GmbH), a company affiliated to the German Trauma Society. The scientific leadership is provided by the Committee on Emergency Medicine, Intensive Care and Trauma Management (Sektion NIS) of the German Trauma Society. The participating hospitals submit their data pseudonymized into a central database via a web-based application. Scientific data analysis is approved according to a peer review procedure laid down in the publication guideline of TR-DGU.

The participating hospitals are primarily located in Germany (90%), but a rising number of hospitals of other countries contribute data as well (at the moment from Austria, Belgium, Finland, Luxembourg, Slovenia, Switzerland, The Netherlands, and the United Arab Emirates). Currently, approx. 38,000 cases from nearly 700 hospitals are entered into the database per year. Participation in TR-DGU is voluntary. For hospitals associated with TraumaNetzwerk DGU^®^, however, the entry of at least a basic data set is obligatory for reasons of quality assurance.

This study was conducted according to the publication guideline of the TR-DGU and registered as project number 2022-018.

Since only patients with severe injury and potentially severe hemorrhage were required for this study all patients documented with the basic dataset only were excluded from analysis, as were all patients with minor injuries. To adequately evaluate prehospital data and resulting initial treatment, we excluded all patients, who were transferred from other hospitals as well as early transfers to other hospitals. Children and adolescents younger than 16 years were also excluded, as we used the ABC- and rSIG-score to estimate the need for early transfusion.

### Patients and inclusion criteria

In the TR-DGU blood transfusion is documented as number of units of packed red blood cells (pRBC) until intensive care unit (ICU) admission. A mass transfusion is defined as 10 + units of pRBC. As this regularly occurs in less than 24 h, a massive transfusion is already present if the 10 units of pRBC required by definition have already been given in a significantly shorter time.

The “Assessment of Blood Consumption” (ABC) score uses four items to predict the need for a massive transfusion in trauma patients: presence of a penetrating injury, initial systolic blood pressure (BP) ≤ 90 mmHg, initial heart rate (HR) > 120 bpm and free fluid in the FAST-exam. Each item receives 0 or 1 point. If the sum value is < 2, a massive transfusion (10 units of pRBC/ 24 h) is unlikely [14]. 

The “rSIG” score is calculated from the “reversed shock index” (systolic blood pressure / heart rate) multiplied by the Glasgow Coma Scale. If the score is ≤ 9.52, the need for a massive transfusion is very likely [11]. 

**Fig. 1:**
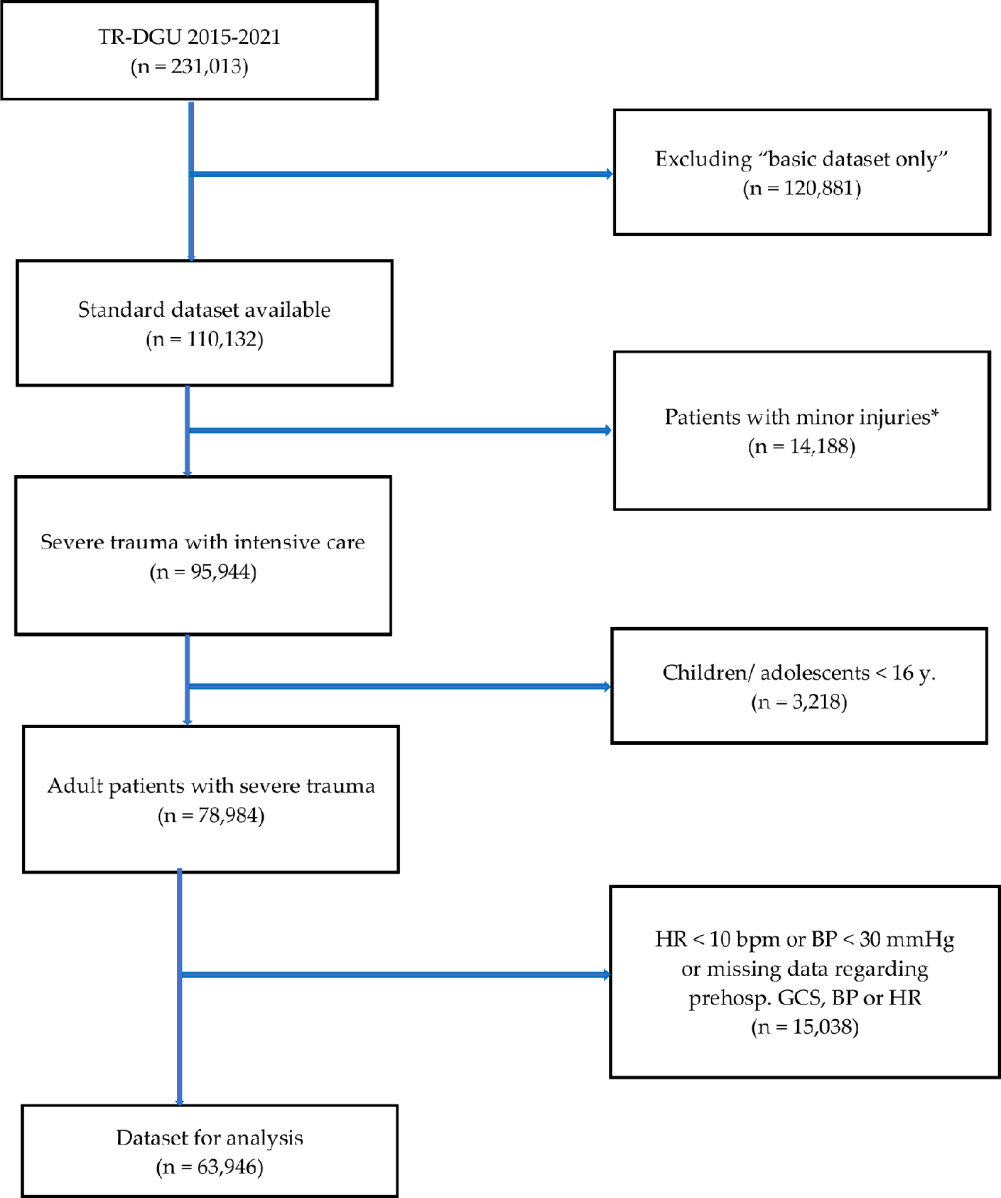
Data selection process: *minor injuries = maximal abbreviated injury scale 1 or 2 without ICU admission

All incomplete datasets regarding prehospital values for GCS, HR, and BP were excluded because those values were needed to calculate the above-mentioned scores. In a last step, all implausible datasets with a documented HR < 10 bpm and a BP < 30 mmHg were excluded (Fig. 1 – data selection process).

Using data of the TR-DGU, the predictive quality of the ABC- and rSIG score for any transfusion (≥ 1 unit pRBC) and mass transfusion (≥ 10 units pRBCs) was calculated. The AUROC for both scores were calculated in relation to the cohort to be able to make comparisons with earlier studies [15]. 

In a further step, the influence of the individual items of the two scores on the predictive quality was determined.

Pseudonymized data collected for the legally required external quality control were used for this analysis; the use of these routinely available data would not require a separate ethics vote.

### Statistics

Categorial data are presented as number of cases and percentage, and metric data are provided as mean with standard deviation (SD). The main endpoints were any blood transfusion (one or more units of packed red blood cells (pRBC) until ICU admission) and mass transfusion (≥ 10 pRBCs until admission to ICU).

Logistic regression analyses were used to quantify the adjusted importance of various predictors. Based on these results the most important results were used to create a new score, the pre-hospital trauma-associated severe hemorrhage (phTASH) score. The area under the receiver operating characteristic (AUROC) with 95% confidence interval was calculated for the existing score systems as well as the newly designed phTASH.

Calculations were performed with SPSS statistical software (version 29.0, IBM Corp., Armonk, NY, USA).

## Results

During 2015–2021 a total of 231,013 datasets coming from a total of 694 hospitals were documented in the TR-DGU From these datasets, we extracted 63,946 datasets for further analysis (Fig. 1).

### Demographics

In the selected collective, the mean patient age was 53.7 years (SD 21.1). It decreased to 48.8 years (SD 11.3) in patients who received a massive transfusion (MT). Male patients accounted for 73.7%. The average ISS was 18.4 (SD 11.3) in the total collective and increased to 39.9 (SD 16.3) in the MT group.

Massive transfusions occurred in a total of 639 patients, which corresponds to about 1% of the analyzed patient collective. A transfusion after trauma occurred in 5,407 cases (8.5%) of the total collective.

Patients with a potential need for MT suffered a traffic accident in 61.3% of cases, with car occupants affected in 22.5%, motorcyclists in 20.7% and pedestrians in 11.1%. Furthermore, falls from a height > 3 m were associated with MT in 17.1% and other causes in 18.5% of cases.

The following body regions were affected when MT was necessary: Thorax 74.2%, extremities 69.0%, abdomen 50.5%, skull 42.7%.

MT was performed in 93.7% of cases in level-I- trauma centers, 5.9% in level-II- trauma centers and 0.3% in local trauma centers.

In 55.5% of cases, the patient was transported to the hospital by ground transport.

### Clinical parameters

The preclinically collected data related to a transfusion or massive transfusion are shown in Table 1 (mean ± SD).

**Table 1:** Prehospital data related to categorized number of administered pRBC (massive transfusion vs any transfusion vs no transfusion)

	No transfusion *N* = 58,539	Any transfusion (≥ 1 pRBC) *N* = 5,407	Massive transfusion (≥ 10 pRBC) *N* = 639
Male gender	41,476 (70.9%)	3,731 (69.0%)	471 (73.7%)
Age [y]	53.6 ± 21.2	52.2 ± 20.9	48.8 ± 19.3
ISS [pts]	17.6 ± 10.4	31.7 ± 14.9	39.9 ± 16.3
Prehospital volume [1]	659 ± 497	1,103 ± 800	1,384 ± 1031
BPsys [mmHg]	138 ± 29	115 ± 33	102 ± 37
Heart rate [bpm]	89 ± 20	101 ± 26	106 ± 29
Shock Index	0.68 ± 0.24	0.97 ± 0.46	1.16 ± 0.54
GCS [pts]	12.7 ± 3.8	10.5 ± 4.8	9.3 ± 5.0
Hospital mortality	5,824 (8.8%)	1,496 (27.7%)	284 (44.4%)

### Influence of items on score prediction

The new designed phTASH score consists of a total of 6 items, which were the most important items in the performed logistic regression analysis for the prediction of a need for transfusion. They can easily be collected prehospitally. Apart from the prediction of a massive transfusion, the predictive value for the need for transfusions from the first pRBC was also calculated, as there are significantly more patients who require a transfusion after trauma in principle, but only rarely receive a mass transfusion.

The items of the phTASH are:

- HR ≥ 120 bpm.
- BPsys ≤ 90 mmHg.
- Shock index > 1.
- GCS ≤ 8.
- Detection of free fluid in the FAST.
- Presence of a penetrating injury.

The influence of individual variables on the prediction result of the phTASH varies in intensity. Table 2 shows the results of the logistic regression analysis of all selected parameters in the order of their influence on the prediction of the need for any or even massive tranfusion.

**Table 2:** Results of the logistic regression analysis representing the influence of each item on the score result

Score-Parameter	Odds Ratio	95% CI for OR	Significance
Positive FAST exam	5.28	4.42–6.32	*P* < 0.001
BPsys < 90 mmHg	2.94	2.35–3.69	*P* < 0.001
SI > 1	2.49	1.93–3.21	*P* < 0.001
GCS ≤ 8	2.28	1.92–2.70	*P* < 0.001
HR > 120 bpm	2.07	1.69–2.53	*P* < 0.001
Penetrating trauma	1.86	1.42–2.42	*P* < 0.001

### phTASH: correlation between score and transfusion probability

The phTASH consists of a total of six individual parameters, which contribute to the score as a simple point value if the respective value is present. By adding up the individual points, the probability of a transfusion or massive transfusion occurring can be derived from the sum. (Table 3).

**Table 3:** phTASH score and associated probability of (massive) transfusion

Points	*n*	Any transfusion (≥ 1 pRBC)	Massive transfusion (≥ 10 pRBC)
0	42,179	3.6%	0.2%
1	13,952	10.6%	1.0%
2	4,479	21.9%	2.6%
3	2,220	38.2%	6.3%
4	928	48.4%	11.5%
5	183	67.8%	21.3%
6	5	100%	40.0%

The risk for transfusion is regarded as “low” (~ 10%), if the socre results in al value of 1–2 points, as “medium” (~ 10–40%) if the score results in2-3 points and “high”(> 40%) if the socre results in ≥ 4 points.

### Score validity

To determine the score predictive quality, the area-under-the-reciever-operating-charecteristics-curve (AUROC) was calculated for ≥ 1 pRBC and ≥ 10 pRBCs respectively.

**Fig. 2:**
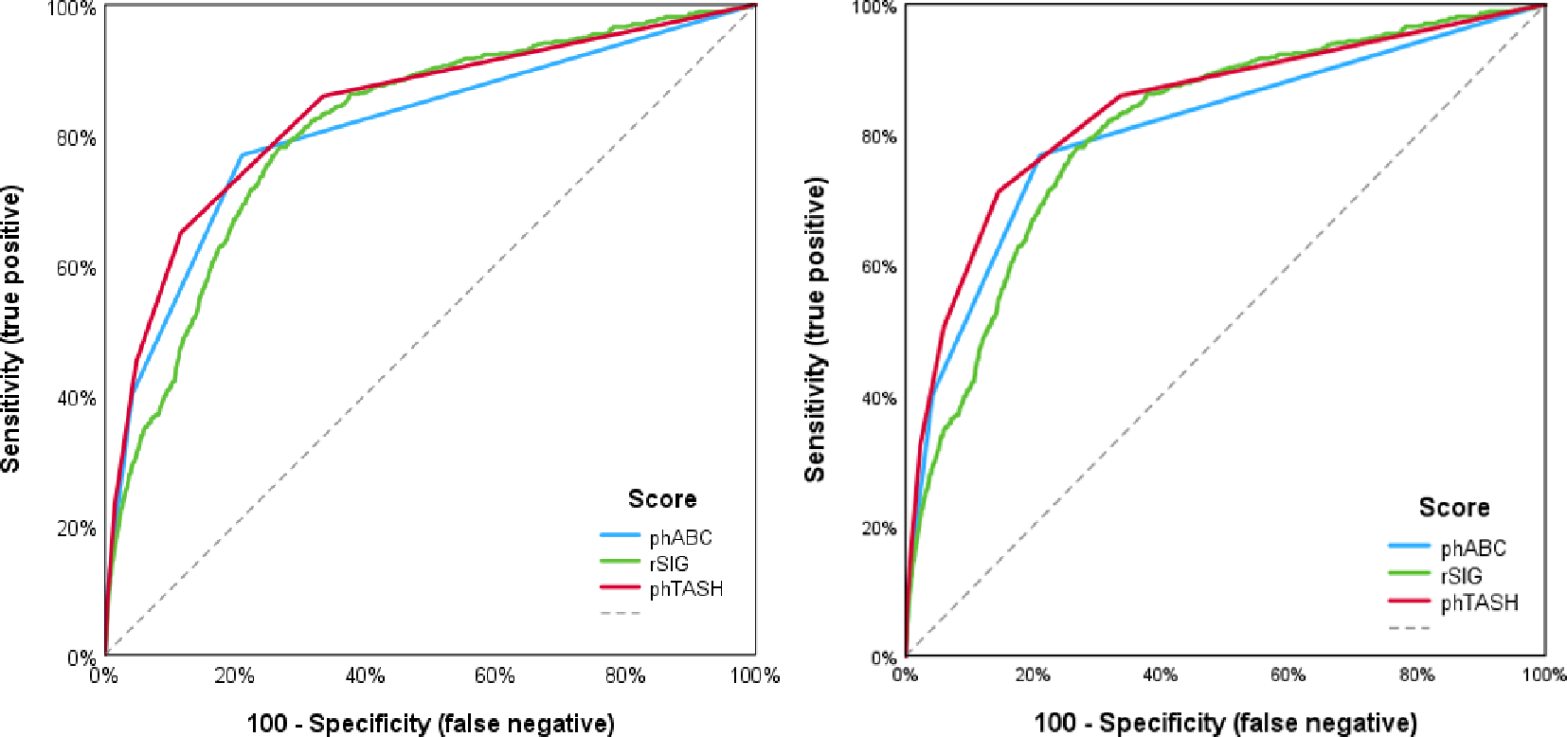
**a**. The ROC curves of all three scores show the differences in prediciton quality for any transfusion (≥ 1 pRBC) until ICU admission) **b**. ROC curves for massive transfusion (≥ 10 pRBCs until ICU admission)

In addition to validation of the predictive quality of the rSIG score and the ABC score with the available data, the combined phTASH score was also subjected to the AUROC analysis (Fig. 2a + b, Table 4).

**Table 4:** AUROC of all scores for transfusion and massive transfusion, with 95% confidence interval in brackets

Score	Prediction of any transfusion	Prediction of massive transfusion
ABC	0.711 (0.702–0.719)	0.806 (0.786–0.826)
rSIG	0.737 (0.730–0.744)	0.807 (0.790–0.824)
phTASH	0.747 (0.739–0.755)	0.834 (0.816–0.852)

## Discussion

In the current study, we could confirm the high predictive quality for the occurrence of massive transfusion of the ABC- and the rSIG-Score also for a mostly German trauma population that correlates more with the European trauma population than military or US data.

Additionally, the phTASH-Score was developed using the items from both scores with the main influencet on the predictive quality resulting in an even higher AUROC than the ABC- and rSIG score provide and is therefore even more accurate. The preclinical FAST-examination seems to be decisive for prediction of (mass-)transfusion as its OR of 5.28 is almost twice of all other items.

In recent decades, the concept of Damage Control Resuscitation has made a decisive contribution to improving the survival of severely injured patients [16, 17]. Based on studies from military and civilian trauma care, therapy with blood products has also become a mainstay in the treatment of patients with hemorrhage after trauma [18, 19]. This is also reflected in various national and international guidelines on trauma care [20, 21]. 

Particularly in the case of battlefield injuries and penetrating injuries in the civilian sector, an advantage in improving early survival has been demonstrated, which has led to an increase in the provision of blood products on rescue helicopters and ambulances in civilian rescue services [1, 22]. 

Therapy with blood products initiated prehospital ensures a significantly earlier start to treatment. This also applies in well-established rescue service systems with relatively short transportation times, as is the case in Germany, for example [23–25]. 

While in the United States the proportion of penetrating injuries is also quite high in civilian emergency medical services, this is not the case in Europe and especially in German-speaking countries (Germany, Austria, Switzerland), where blunt trauma is the leading cause of injury [26, 27]. 

In principle, blood products can also be used in a resource-saving manner in the prehospital setting [23]. However, the uncritical use of blood products poses a problem, as in addition to the unnecessary use of a scarce resource, the administration of blood products is also associated with a potential outcome disadvantage for the patient due to an infection/procedure risk (transfusion reactions) [28]. 

To increase the confident use of blood products several scores were examined with regard to their predictive quality for (massive) transfusion in the context of severe trauma. However, clinical scoring systems cannot be used in the prehospital setting due to unavailable resources including laboratory tests [9, 15]. 

However, the prehospital stage plays a decisive role in the care of severely injured patients. In fact, therapeutic steps will be initiated within the “golden hour of trauma” that significantly influence the outcome of the patients. Furthermore, an anticipatory prehospital approach may further decrease time to transfusion in hemorrhagic shock further reducing cell death and organ failure. Moreover, not only is the assessment of an appropriate receiving hospital, but also adequate and reproducible advance notification of the patient’s condition can ensure optimal preparation of the hospital to enable seamless continuation of therapeutic efforts.

Since many of the available scores have been validated using heterogeneous patient populations that are not directly transferable to Europe [7, 29] and other scores that are based upon European data allow only an indirect assessment of massive bleeding [30] we first calculated the predictive quality for our own patient population. By estimating the influence of individual score parameters on the prediction quality, it appears that both the ABC score and the rSIG score each contain individual items that have a major influence on the prediction but are not considered in the other score, which is why the problem was solved in this study by combining the items of both scores.

This new score for the preclinical assessment of hemorrhage after severe trauma (phTASH) is easy to collect preclinically and has because of its higher AUROC a better predictive quality than the ABC and rSIG score investigated, both for the prediction of a transfusion in general and a possible massive transfusion.

The phTASH score is easy to calculate which is a major difference to the rSIG score which makes it more failure proof in stressful situations. The phTASH data show that a score value of 0–1 represents a low probability of transfusion, a score value of 2–3 represents a medium probability of transfusion and a score value of ≥ 4 points represents a high risk of transfusion and massive transfusion.

As the TR-DGU only registers the number of transfused pRBCs up to admission to the intensive care unit, the definition of massive transfusion is fulfilled in a much narrower period of time (< 24 h).

A closer look at the influence of individual items on the probability of transfusion shows that, the positive FAST exam, defined as free fluid in the abdomen, has the greatest influence within all items of the score.

To our knowledge, this study is the first to clearly demonstrate the high diagnostic value of preclinical ultrasound, which remains controversial in the literature [29, 31]. 

All rescue assets in the German Emergency medical system (EMS) that carry blood products are equipped with ultrasound devices. The emergency physicians on these vehicles and helicopters have also received training in emergency sonography during their specialist training before being deployed as emergency physicians. The quick performance of an ultrasound examination can be easily integrated into the rescue mission by well-rehearsed teams, without losing additional time on scene, and thus delaying transport to the hospital. Often, the prehospital ultrasound is performed as adjunct to the primary survey while the EMS crew member coordinates with the designated hospital and prepares the transport.

Regardless of this, it must be demanded that all healthcare providers in charge on site are adequately trained to perform an emergency ultrasound examination in order to achieve reliable examination results and to be able to reliably include or exclude pathologies. A possible training concept was already published in 2013 by Press et al. [32]

The data support the applicability of the score to the German-speaking/European area, as penetrating injuries have the least influence on the result compared to the other items of the score.

The data also show that a large loss of blood into the large cavities must be expected, especially in the context of traffic accidents and falls from a great height.

A limiting factor of the presented study is that it is a retrospective analysis of register data. Furthermore, preclinically collected vital signs had to be combined with clinical values (e.g. free fluid in the abdomen in the FAST) to calculate the score values. Furthermore, the correlation with clinically performed transfusions was carried out without the individual transfusion trigger being documented in the individual data set.

As we excluded mild injuries, the predictive quality of the phTASH score is yet unknown.

Furthermore, the phTASH score implemented by us still must be subjected to external validation, e.g. by applying it to data from other databases.

## Conclusions

The early use of blood products in the context of severe hemorrhage is part of guideline-compliant therapy. The phTASH score presented here offers the best predictive quality to date for a valid prognostication of the patient’s need for transfusion at the prehospital stage with limited diagnostic options.

Preclinical ultrasound has a firm place in the adequate assessment of severe trauma.

Further prospective studies on the influence of early application of blood products on patient survival are necessary to demonstrate the preclinical benefit.

The new phTHAS score will be implemented to assess the prehospital need for transfusion in several German HEMS stations. A further proliferation on other EMS plattforms is also planned for the future.

## Data Availability

The data that support the findings of this study are available from DGU but restrictions apply to the availability of these data, which were used under license for the current study, and so are not publicly available. Data are however only available from the authors upon reasonable request and with permission of DGU.

## Abbreviations

ABC score: Assessment of Blood Consumption score
AUC: Akademie für Unfallchirurgie – company affiliated with the German Trauma Society
AUROC: Area under the receiver operating characteristics curve
BP (sys): Blood pressure (systolic)
CI: Confidence intervall
DGU: Deutsche Gesellschaft für Unfallchirurgie (German Trauma Society)
EMS: Emergency Medical Service
FAST exam: Focused Assessment with sonography for trauma
GCS: Glasgow Coma Scale
HEMS: Helicopter Emergency Medical System
HR: Heart rate
ICU: Intensive care unit
ISS: Injury severity score
MAIS: Maximal abbreviated injury scale
MT: Massive transfusion
OR: Odds ratio
phTASH: prehospital Trauma Associated Severe Hemorrhage
pRBC: Packed red blood cells
rSIG score: Reversed Shock Index x Glasgow Coma Scale score
SD: Standard deviation
Sektion NIS: Committee on Emergency Medicine, Intensive Care and Trauma Management of the DGU
SI: Shock index
TR-DGU: TraumaRegister der DGU – trauma registry oft he German Trauma Society
